# ECHO-T2D - economic, clinical epidemiologic, and humanistic profiling of diabetic retinopathy, diabetic kidney disease, and painful diabetic peripheral neuropathy in India: A systematic review protocol

**DOI:** 10.1016/j.mex.2026.103953

**Published:** 2026-05-12

**Authors:** Muhammad Aaqib Shamim, Krishna Tiwari, Ravindra Shukla, Mukesh Kumar Swami, Shival Srivastav, Seema Meena, Manish Chaturvedy, Samhita Panda, Denny John, Muhammad Aasim Shamim, Shoban Babu Varthya, Surjit Singh, Pradeep Dwivedi

**Affiliations:** a Department of Pharmacology, All India Institute of Medical Sciences, Basni Industrial Area, MIA 2nd Phase, Basni, Jodhpur, Rajasthan 342005, India; b Department of Endocrinology & Metabolism, All India Institute of Medical Sciences, Basni Industrial Area, MIA 2nd Phase, Basni, Jodhpur, Rajasthan 342005, India; c Department of Psychiatry, All India Institute of Medical Sciences, Basni Industrial Area, MIA 2nd Phase, Basni, Jodhpur, Rajasthan 342005, India; d Department of Physiology, All India Institute of Medical Sciences, Basni Industrial Area, MIA 2nd Phase, Basni, Jodhpur, Rajasthan 342005, India; e Department of Ophthalmology, All India Institute of Medical Sciences, Basni Industrial Area, MIA 2nd Phase, Basni, Jodhpur, Rajasthan 342005, India; f Department of Nephrology, All India Institute of Medical Sciences, Basni Industrial Area, MIA 2nd Phase, Basni, Jodhpur, Rajasthan 342005, India; g Department of Neurology, All India Institute of Medical Sciences, Basni Industrial Area, MIA 2nd Phase, Basni, Jodhpur, Rajasthan 342005, India; h Adjunct Professor, School of Public Health, University of Adelaide, Adelaide, Australia; i Institute of Health Economics Epidemiology and Translational Science (IHEETS), Thiruvananthapuram, Kerala, India; j Prasanna School of Public Health, Manipal Academy of Higher Education (MAHE), Manipal, Karnataka, India; k Department of Hospital Administration, Post Graduate Institute of Medical Education and Research, Chandigarh 160012, India; l Centre of Excellence for Tribal Health, All India Institute of Medical Sciences, Basni Industrial Area, MIA 2nd Phase, Basni, Jodhpur, Rajasthan 342005, India

**Keywords:** Markov model inputs, Pharmacoeconomic model inputs, Integrated review, Microvascular complications, T2D

## Abstract

Microvascular complications of type 2 diabetes (T2D) like diabetic retinopathy (DR), diabetic kidney disease (DKD), and painful diabetic peripheral neuropathy (pDPN) contribute to morbidity, impaired quality of life, and healthcare costs. Evidence describing their clinical course, economic burden, and humanistic impact remains fragmented, limiting its utility for policy and health economic modelling.

We aim to carry out an integrated synthesis of the economic, clinical epidemiologic, and humanistic outcomes associated with DR, DKD, and pDPN among individuals with T2D in India. All primary studies on our research question are eligible. Economic outcomes include both costs and resource consumption. Clinical epidemiologic outcomes cover disease staging, severity markers, and progression. Humanistic outcomes include health-related quality of life with utility and disutility values.

Our results will provide consolidated and methodologically justified, model-ready estimates of clinical trajectories, economic burden, and health-related quality of life that are specific to a local context and can be adapted elsewhere. These outputs will strengthen health economic evaluations by reducing reliance on non-contextual data. In turn, the evidence generated will support the updating of clinical practice guidance, identify key gaps in the existing evidence base to guide future research, and inform priority setting for resource allocation within constrained healthcare resources.

## Graphical abstract

**Figure:**
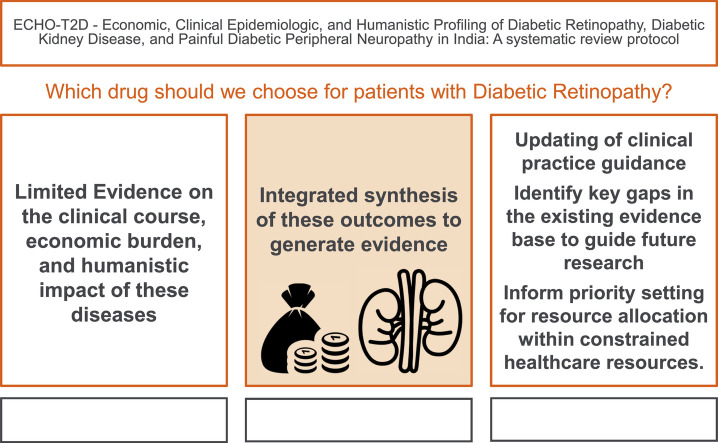

## Specifications table

**Table:** 

**Subject area**	Medicine and Dentistry
**More specific subject area**	Evidence Synthesis; Health Economics
**Name of your protocol**	ECHO-T2D - Economic, Clinical Epidemiologic, and Humanistic Profiling of Diabetic Retinopathy, Diabetic Kidney Disease, and Painful Diabetic Peripheral Neuropathy in India: A systematic review protocol
**Reagents/tools**	Not applicable
**Experimental design**	Protocol to a Systematic Review
**Trial registration**	Not applicable
**Ethics**	This protocol involves already collected data, and is exempt from ethics as per “Ethical Requirements for Systematic Review & Meta-Analysis Proposals” by a 2024 addendum to Indian Council of Medical Research National Ethical Guidelines for Biomedical and Health Research Involving Human Participants, 2017
**Value of the Protocol**	• Our results will provide consolidated and methodologically justified, model-ready estimates of clinical trajectories, economic burden, and health-related quality of life that are specific to a local context and can be adapted elsewhere. • These outputs will strengthen health economic evaluations by reducing reliance on non-contextual data. • In turn, the evidence generated will support the updating of clinical practice guidance, identify key gaps in the existing evidence base to guide future research, and inform priority setting for resource allocation within constrained healthcare resources.

## Background

Complications of Type 2 Diabetes (T2D) account for much of the long-term morbidity and mortality associated with the disease and drive a substantial portion of both direct and indirect healthcare costs [[1], [2], [3]]; and affects over 100 million individuals in the country [4] Some chronic microvascular sequelae of T2D, specifically diabetic retinopathy (DR), diabetic kidney disease (DKD), and painful diabetic peripheral neuropathy (pDPN), represent a triad of conditions that impair quality of life and impose considerable financial hardship on patients and health systems. Prevalence studies from India report that microvascular complications are common among people with diabetes, with diabetic retinopathy estimates ranging up to one in every sixth patient [5]

Existing evidence on economic burden in India demonstrates that diabetes and its complications impose significant financial strain on affected households. Studies of diabetes cost of illness in India describe substantial expenditures, yet these are often fragmented, limited in scope, and rarely disaggregated by specific complications, particularly microvascular conditions. Furthermore, existing cost studies frequently do not comprehensively quantify humanistic outcomes, such as health-related quality of life (QoL) or utility values, that are essential for a comprehensive assessment and for parameterizing economic evaluations [6,7]

While individual studies may report some of prevalence, clinical trajectories, cost estimates, or quality of life outcomes for DR, DKD, or pDPN in India, there remains a substantial gap in evidence synthesis that simultaneously integrates the clinical epidemiologic, economic, and humanistic dimensions of these microvascular complications [[8], [9], [10], [11], [12], [13], [14], [15]] This lack of integrated evidence is especially meaningful in India where health system delivery varies widely, patterns of healthcare utilization differ across regions, and a high reliance on out-of-pocket payments can influence both access to care and the distribution of economic burden [[16], [17], [18], [19]]

Decision-analytic models, including Markov cohort models and discrete event simulation, are widely used to estimate the long-term cost-effectiveness of drugs and other health strategies over relevant time horizons [[20], [21], [22]] The validity and policy relevance of these models depend on the accuracy and appropriateness of input parameters, including disease transition probabilities, health state utilities, resource utilization patterns, and costs specific to the population and health system [23] Inputs derived from surrogate or high-income country contexts may not accurately represent local Indian epidemiology, cost structures, or patient preferences, thereby limiting model validity and potentially introducing significant bias in economic evaluation outcomes [24] Downstream of these decision-analytic models, the results dictate policy decisions[[25], [26], [27], [28], [29]] and priority setting[30,31]. Moreover, systematic aggregation and critical appraisal also clarifies the quality, scope, and limitations of existing data and help identify priority areas for new primary research.

By systematically profiling economic costs (direct and indirect), clinical epidemiologic characteristics (including disease staging and progression), and humanistic outcomes (such as utility and disutility) for DR, DKD, and pDPN specifically within the Indian population, we seek to fill these evidence gaps and provide a structured foundation to support more efficient allocation of constrained health resources and ultimately contribute to improved health outcomes for people living with diabetic complications in India.

## Description of protocol

### Reporting

We have developed and reported this systematic review protocol considering Preferred reporting items for systematic review and meta-analysis protocols (PRISMA-P[32]), and registered the study protocol at the Open Science Framework [https://doi.org/10.17605/OSF.IO/DEJFV] [33].

### Research question

We aim to synthesise the evidence on the economic, clinical epidemiologic, and humanistic outcomes associated with three microvascular complications (DR, DKD, and PDPN) of T2D in India. Accordingly, our research question is on the population of DR, DKD, and PDPN of T2D, address the economic, clinical epidemiologic, and humanistic outcomes, and focuses on an Indian context, across all healthcare settings.

### Eligibility

We will include all studies involving individuals diagnosed with T2D and with either of these three microvascular complications - DR, DKD, and PDPN. Studies will be eligible regardless of participants’ age, sex, socioeconomic status, disease duration, or severity, provided the population is based in India.

Eligible studies must report at least one outcome of interest within one or more of the following domains: economic, clinical epidemiologic, or humanistic outcomes. Economic outcomes will capture the financial burden and resource utilisation associated with these conditions. Extracted data will include the currency and price year, as well as the economic perspective adopted by the study. Costs will be classified into direct medical, direct non-medical, and indirect costs. Direct medical costs will include expenditures related to outpatient and inpatient consultations, diagnostic investigations, pharmacotherapy, and complication-specific treatments such as laser photocoagulation, intravitreal injections, dialysis, or other procedure-based care. Direct non-medical costs will encompass patient- or caregiver-incurred expenses such as transportation, food, lodging, and costs related to lifestyle modifications necessitated by disease management. Indirect costs will include productivity losses, wage loss for patients and caregivers, and other measures of income loss attributable to morbidity or care requirements. Where reported, total economic burden, including aggregated direct and indirect costs and average annual costs, will be extracted.

Clinical epidemiologic outcomes will characterise the epidemiology, severity, and progression of each condition. For DR, extracted outcomes will include disease prevalence, diagnostic methods, visual status, treatment history, and complication-specific risk factors. For DKD, outcomes will include prevalence, renal function markers, management status, dialysis frequency, and associated complications. For pDPN, outcomes will include disease prevalence, diagnostic tools, and pain severity measures. Across conditions, clinical epidemiologic data related to disease staging, severity indicators, and progression patterns will be synthesised when available. Humanistic outcomes will assess the impact of these complications on patient and caregiver well-being. Extracted data will include the quality-of-life instrument used, reported scores, and key findings, including comparisons across disease stages or populations when available. Utility and disutility values derived from validated instruments will be extracted to support health economic evaluations.

All primary studies reporting relevant outcome data will be eligible for inclusion. Case reports, case series, modelling studies based solely on secondary data, non-human studies, non-English publications, and studies not conducted in Indian populations will be excluded.

### Search

We will search PubMed, Embase, and the Cochrane Central Register of Controlled Trials from database inception till 30th November 2025. The search strategy will incorporate index terms and controlled vocabularies informed by existing search filters[34] and prior systematic reviews[[35], [36], [37], [38], [39], [40]] addressing parts of the research question. Search strategies are provided in Annexure 1. To enhance coverage of locally relevant evidence and grey literature, the Shodhganga database will be searched which is an Indian repository of theses and dissertations. In addition, subject experts will be contacted, and reference lists of included studies will be screened to identify further eligible publications.

### Study selection

All records retrieved from the searches will be collated, and duplicates will be removed prior to screening. Titles and abstracts will be screened, followed by full-text assessment of potentially eligible studies. Screening will be conducted initially on a set of records reviewed independently by two human reviewers to train a robot screener, and the remaining records will then be screened by one reviewer in conjunction with this trained robot screener. The robot screener will also be iteratively refined through conflict resolution [41] Final inclusion decisions will be made by a human reviewer to ensure oversight. Disagreements will be resolved through discussion or, when necessary, consultation with a senior review author. Reasons for exclusion at the full-text stage will be documented and reported in the supplementary material.

### Risk of bias assessment

Risk of bias will be assessed using tools appropriate to the type of data reported. Studies reporting economic outcomes will be appraised using Consensus on Health Economic Criteria[42] and Consolidated Health Economics Evaluation Reporting Standards[43]. Clinical epidemiologic outcome studies will be assessed using the revised Risk of Bias 2 tool[44], or other study design-specific critical appraisal tools recommended by JBI [45] Humanistic outcome data will be appraised using a two-tiered approach: the psychometric validity of the instruments themselves will be evaluated using COnsensus-based Standards for the selection of health status Measurement Instruments [COSMIN][46], while the risk of bias for these outcomes within primary studies will be assessed using the Cochrane RoB 2 tool or other study design-specific critical appraisal tools recommended by JBI. Modelling studies, where applicable, will be assessed for the extent of model validation. Risk-of-bias assessments will be conducted using structured templates adapted from prior work[36], with an example for economic evaluations provided in Annexure 2.

### Data extraction

A standardised data extraction form has been developed for consistent and comprehensive capture of relevant information. Data extraction fields are organised into four domains. General study characteristics include study design, publication year, geographic location within India, clinical setting, and sample size. Participant-level information include age, sex distribution, socioeconomic status, and duration of T2D [Annexure 3].

Economic data will be extracted to characterise financial burden, including direct medical costs, direct non-medical costs, and indirect costs. Specific cost components will include consultations, diagnostic investigations, pharmacotherapy, and complication-specific interventions such as dialysis and laser photocoagulation. Information on currency, price year, and economic perspective will also be recorded [Annexure 4]. Clinical epidemiologic data extraction will capture disease staging, severity measures, and progression indicators specific to each complication [Annexure 5]. Humanistic outcomes will include the tools used to measure health-related quality of life, reported utility and disutility values, and relevant domain-level scores [Annexure 6]. Detailed extraction templates for each outcome domain are provided in Annexures C-F.

Two reviewers will extract data from included studies, resolving discrepancies through discussion or consultation with a third reviewer. When required, corresponding authors will be contacted to obtain missing or unclear information.

### Data synthesis

Economic, clinical epidemiologic, and humanistic outcomes will be synthesised in a unified framework. Depending on the nature and comparability of the available data, findings may be tabulated narratively and synthesised without meta-analysis[47], or if appropriate[48], quantitatively synthesised. Meta-analyses will be conducted separately for each of DR, DKD, and pDPN, and further stratified by outcome domain to preserve conceptual coherence. For prevalence-type outcomes, including overall prevalence of each microvascular complication, disease stage distributions, and complication-specific frequencies, pooled proportions will be estimated using suitable transformations. Summary prevalence estimates will be back-transformed and presented as proportions. For continuous clinical measures such as renal function indices, visual acuity scores, or pain severity scores, pooled estimates will be calculated using mean differences when outcomes are reported on the same scale, and standardised mean differences or ratio of means when different instruments are used to measure comparable constructs.

Economic outcomes will be meta-analysed only when feasible [49] These will be synthesised separately by cost category, including direct medical, direct non-medical, and indirect costs. Prior to pooling, all cost data will be standardised to a common price year and converted to international dollars using purchasing power parity deflators for comparability [50] When reported cost distributions are skewed, log-transformation will be considered prior to meta-analysis, and pooled estimates will be back-transformed for presentation. Humanistic outcomes, including health-related quality of life and utility values, will be synthesised by measurement instrument and outcome domain. Where studies report associations between disease severity and quality-of-life outcomes, these relationships will be summarised quantitatively or narratively depending on reporting consistency. Utility decrements associated with advancing disease stages will also be extracted and pooled.

Acknowledging expected heterogeneity, random-effects meta-analysis models will be used, and all summary estimates will be reported with 95 percent confidence intervals and 95 percent prediction intervals[51,52]. Statistical heterogeneity will be assessed using prediction intervals, I-squared, and tau-squared statistics. Subgroup analyses will explore variation by disease stage (such as PDR or NPDR), healthcare settings, where sufficient data are available. Sensitivity analyses will assess the robustness of findings to alternative case definitions and exclusion of atypical studies. Publication bias will be evaluated using funnel plots and Egger regression[53] when at least ten studies are available, while Doi plots with the LFK index[54] will be used when at least five studies will be available or single-group data will be reported. Assessment of reporting bias will extend comprehensively beyond publication bias alone [55]

### Confidence in cumulative evidence

The certainty of evidence for the synthesised outcomes will be assessed using the GRADE (Grading of Recommendations Assessment, Development, and Evaluation) framework [56] Summary of findings tables will be used to present certainty assessments.

### Protocol validation

Not applicable.

### Limitations

We acknowledge some limitations. First, the review is dependent on the availability and quality of primary studies, which may vary in methodological rigour, reporting standards, and outcome definitions. This will be addressed through structured risk-of-bias assessment using domain-appropriate tools and transparent reporting of evidence certainty. Second, heterogeneity in disease severity classifications, costing approaches, and quality of life instruments may limit the feasibility of quantitative synthesis or direct comparability across studies. Where this occurs, findings will be synthesised narratively with justifications. Third, gaps in published data, particularly for humanistic outcomes and long-term cost trajectories, may restrict the availability of certain model parameters. These gaps and limitations wherever obtained will be explicitly documented, and their implications will be highlighted to guide future data generation.

Our protocol focuses on the integration of humanistic outcomes, which are frequently underreported in clinical and economic studies but are central to comprehensive burden assessment. Measures of health-related quality of life and utility values are essential for cost-utility analyses and for understanding the broader impact of chronic complications on patients and caregivers, particularly in long-term conditions where symptom burden and functional impairment strongly influence care decisions.

This systematic review protocol aims to generate an integrated and contextually relevant evidence base on the economic, clinical epidemiologic, and humanistic burden of diabetic retinopathy, diabetic kidney disease, and painful diabetic peripheral neuropathy in India. Subsequently, this review seeks to support evidence-informed clinical practice, strengthen health economic modelling, and contribute to more efficient allocation of constrained health resources, ultimately improving outcomes for people living with type 2 diabetes in India.

## CRediT author statement

MAqS: Conceptualization, Methodology, Project administration, Writing – original draft, Writing – review & editing. KT: Conceptualization, Methodology, Project administration, Writing – original draft, Writing – review & editing. RS: Conceptualization, Methodology, Project administration, Writing – original draft, Writing – review & editing. MKS: Conceptualization, Methodology, Project administration, Writing – original draft, Writing – review & editing. ShSr: Conceptualization, Methodology, Project administration, Writing – original draft, Writing – review & editing. SM: Conceptualization, Methodology, Project administration, Supervision, Writing – review & editing. MC: Conceptualization, Methodology, Project administration, Supervision, Writing – review & editing. SP: Conceptualization, Methodology, Project administration, Supervision, Writing – review & editing. DJ: Conceptualization, Methodology, Project administration, Writing – original draft, Writing – review & editing. MAsS: Conceptualization, Methodology, Project administration, Supervision, Writing – original draft, Writing – review & editing. SBV: Conceptualization, Methodology, Project administration, Supervision, Writing – original draft, Writing – review & editing. SS: Conceptualization, Methodology, Project administration, Supervision, Writing – original draft, Writing – review & editing. PD: Conceptualization, Methodology, Project administration, Supervision, Writing – original draft, Writing – review & editing.

## Declaration of competing interest

The authors declare that they have no known competing financial interests or personal relationships that could have appeared to influence the work reported in this paper.

## Data Availability

No data was used for the research described in the article.

## Appendix Supplementary materials

Image, application 1
